# [Expression of Concern] RIP kinase-mediated ROS production triggers XAF1 expression through activation of TAp73 in casticin-treated bladder cancer cells

**DOI:** 10.3892/or.2026.9192

**Published:** 2026-09-14

**Authors:** Yoon Hee Chung, Daejin Kim

Oncol Rep 36: 1135–1142, 2016; DOI: 10.3892/or.2016.4895

Following the publication of this paper, it was drawn to the Editor's attention by a concerned reader that the JNK western blotting data shown in Fig. 2E on p. 1138 were strikingly similar to the JNK western blotting data shown in Fig. 4D on p. 1139, even though the experimental conditions reported for these figure parts were apparently different.

The authors have been contacted by the Editorial Office to offer an explanation for the apparent re-use of the same data in this paper, and the authors have replied to explain that these studies were performed several years ago, that they no longer have access to the original data, and moreover, that they are not in a position to repeat any of these experiments. Owing to the fact that the Editorial Office has been made aware of potential issues surrounding the scientific integrity of this paper, we are issuing an Expression of Concern to notify readers of this potential problem associated with the abovementioned data.

